# Intravenous fibrinolysis plus endovascular thrombectomy versus direct endovascular thrombectomy for anterior circulation acute ischemic stroke: clinical and infarct volume results

**DOI:** 10.1186/s12883-019-1341-3

**Published:** 2019-05-29

**Authors:** Massimo Gamba, Nicola Gilberti, Enrico Premi, Angelo Costa, Michele Frigerio, Dikran Mardighian, Veronica Vergani, Raffaella Spezi, Ilenia Delrio, Andrea Morotti, Loris Poli, Valeria De Giuli, Filomena Caria, Alessandro Pezzini, Roberto Gasparotti, Alessandro Padovani, Mauro Magoni

**Affiliations:** 1 Stroke Unit, Neurologia Vascolare, ASST Spedali Civili di Brescia, Piazzale Spedali Civili 1, Brescia 25123 Brescia, Italy; 2 Servizio di Neuroradiologia, Università degli Studi di Brescia, Brescia, Italy; 3 Dipartimento di Scienze Mediche e Chirurgiche, Clinica Neurologica, Università degli Studi di Brescia, Brescia, Italy; 4 Stroke Unit, IRCCS Fondazione Istituto Neurologico Nazionale “C. Mondino”, Pavia, Italy

**Keywords:** Ischemic stroke, Intravenous thrombolysis, Endovascular therapy, Combined therapy, Large vessels occlusion

## Abstract

**Background:**

endovascular therapy (ET) is the standard of care for anterior circulation acute ischemic stroke (AIS) caused by large vessel occlusion (LVO). The role of adjunctive intravenous thrombolysis (IVT) in these patients remains unclear. The present study aims to investigate whether IVT followed by ET (CoT, combined therapy) provides additional benefits over direct ET for anterior circulation AIS with LVO.

**Methods:**

we achieved a single center retrospective study of patients with AIS caused by anterior circulation LVO, referred to our center between January 2014 and January 2017 and treated with ET. Functional recovery (modified Rankin at 3-months follow-up), recanalization rate (thrombolysis in cerebral infarction [TICI] score) and time, early follow-up brain CT scan infarct volume (EFIV) (for recanalized patients only), symptomatic intracerebral hemorrhage (sICH) and 3-month mortality were the outcomes of interests. Independent predictors of the outcomes were explored with multivariable logistic regression.

**Results:**

145 subjects were included in the study, of whom 70 underwent direct ET and 75 were treated with CoT. Functional independence at 3-months was more frequent in CoT subjects compared to patients who received direct ET (mRS score 0–1: 48.5% vs 18.6%; *P* < 0.001. mRS score 0–2: 67.1% vs 37.3%; *P* < 0.001); CoT patients had also higher first-pass success rate (62.7% vs 38.6%, *P* < 0.05), higher recanalization rate (84.3% vs 65.3%; *P* = 0.009) and, in recanalized subjects, smaller EFIV (16.4 ml vs 62.3 ml; *P* = 0.003). Mortality and intracranial bleeding did not differ between the two groups. In multivariable regression analysis, low baseline NIHSS score (*P* < 0.05), vessel recanalization (*P* = 0.05) and CoT (*P* = 0.03) were independent predictors of favorable outcome at three months.

**Conclusions:**

CoT appears more effective than ET alone for anterior circulation AIS with LVO, with similar safety profile.

## Background

Endovascular therapy (ET) represents the standard of care for anterior circulation acute ischemic stroke (AIS) due to large vessel occlusion (LVO) [1–3]. It remains unclear whether intravenous thrombolyisis preceding ET provides additional benefit compared to direct ET. The administration of IVT may have important drawbacks such as potential risk of bleeding, especially in patients taking single or dual antiplatelet therapy, delay in beginning of ET and clot fragmentation leading to distal arterial embolism [4]. Conversely, the use of tissue plasminogen activator (tPA) may produce recanalization by itself or may aid thrombectomy by softening the thrombus and enhance overall reperfusion by accelerating lysis of distal thrombi, preserving microvascular perfusion downstream to the arterial occlusion [4]. Recent observational studies and 2 meta-analyses showed conflicting results [5–9]. A large registry, including 599 direct ET and 567 CoT therapy patients found no difference between the 2 groups in terms of safety and efficacy [10]. Another registry of 276 (138 in each group) tPA-eligible patients within a 4.5-h time window, again found no significant difference between CoT and ET for both safety and efficacy parameters [11]. Since the currently available evidence on this topic is inconclusive, 4 randomized clinical trials are ongoing (SWIFT DIRECT, NCT03469206; MR CLEAN NOIV, http://www.mrclean-noiv.nl, DIRECT MT, NCT03469206; DIRECT SAFE NCT03494920) but no one has been published to date.

The goal of our study was to compare CoT versus ET in a real world single center cohort of AIS patients with LVO, exploring the safety profile of these two treatments and comparing their effect on radiological and functional outcomes [12–14].

## Methods

***Subjects.*** single-center retrospective observational study. All AIS patients referring to our center (Stroke Unit, Neurologia Vascolare, ASST “Spedali Civili”, Brescia, Italy) between January 2014 and January 2017 were screened for the study. Subjects with anterior circulation AIS due to LVO and fulfilling AHA/ASA criteria for ET [3] were included in the analysis. LVO was diagnosed by computed tomography angiography as an occlusion involving intracranial terminal internal carotid artery (tICA) and/or M1–proximal M2 tracts of middle cerebral artery (MCA). Patients fulfilling criteria for IVT and ET were allocated to CoT group while subjects with IVT exclusion criteria were included in ET group [3].

Patients treated with CoT received intravenous full-dose rtPA (0.9 mg/kg) followed by ET, with groin puncture performed at the same time of rtPA bolus or as soon as possible during IVT infusion. Patients with undetermined time of symptoms onset and those with ET performed after complete tPA administration were excluded. Written informed consent was obtained by patients or relatives. All the procedures of the study conformed to the Helsinki Declaration.

Demographics, vascular risk factors, laboratory exams, imaging findings and vital signs were collected. Stroke etiology, according to Trial of ORG 10172 in acute stroke treatment (TOAST) criteria 15, was assessed. All patients underwent a baseline brain computed tomography (CT) with Alberta Stroke Program Early Computed Tomography Score (ASPECTS) evaluation [15] and a follow up brain CT at 2–4 days from onset. Baseline National Institute of Health Stroke Scale (NIHSS) were recorded. The number of passes during ET have been recorded as well and the recanalization degree has been assessed on digital subtraction angiography (DSA) according to TICI criteria; Good recanalization was defined as TICI grade 2b or 3 [16].

The main safety outcomes of interest were: symptomatic intracranial hemorrhage (sICH) defined as deterioration in NIHSS ≥4 associated with evidence of any intracerebral hemorrhage on follow-up non-contrast cerebral CT, according to European Cooperative Acute Stroke Study II (ECASS II) [17] and mortality at 3 months follow up.

### Cerebral infarct volume measurement

The Cerebral Infarct Volume (CIV) of all recanalized patients were manually delineated by one experienced neurologist (N.G.) on early follow-up brain CT scan (at 2–4 days after stroke). The infarct volume was delineated section-by-section by using ITK-SNAP 2.2.0 (http://www.itksnap.org/pmwiki/pmwiki.php). Brain CT scan with 5 mm thick slices were used, considering acute cerebral infarct as new parenchymal hypodensities with respect to basal brain CT. Infarct volume was measured manually contouring the parenchymal hypodensity of interest slice by slice. Software then calculated the volume of the selected area [12, 13]. Observer was blinded to all clinical information and outcome.

### Interventional procedures

All procedures were performed under conscious sedation on a biplane angiography (Axiom Artis, Siemens, Erlangen, Germany) avoiding general anesthesia, if possible. Endovascular procedures consisted in thrombectomy with stent retrievers (Solitaire stent-Ev3 Inc. and Trevo stent–Stryker) or thromboaspiration (Penumbra 5 Max, Penumbra, Alameda, California, USA).

### Statistical analysis

Univariate comparisons between the groups were made using Pearson’s chi-squared test for categorical variables, the Mann-Whitney U test or Wilcoxon rank sum test for continuous variables. We assessed both clinical (mRS at 90 days) and neuroradiological outcome (early follow-up Infarct Volume – EFIV - at 2–4 days CT scan, in well recanalized cases). Independent predictors of the outcomes of interest were explored with a multivariable forward stepwise binary logistic regression model. Variables known to be predictive of clinical outcome from the literature were entered into the initial model. SPSS package (v. 17.0, Chicago, IL, USA) was used for the analyses and *p* values < 0,05 were considered statistically significant.

## Results

A total of 2248 AIS patients referred to our Hospital were screened and 145 subjects met the inclusion criteria of our study (70 in CoT and 75 in direct ET group). There was no significant difference between groups regarding age, gender, blood pressure, blood glucose, coronary disease, hypercholesterolemia and antithrombotic medications before stroke. 2 subjects were excluded because they were transferred to our Centre by HUB&SPOKE mechanism late after (> 60 min: 70 and 75 min respectively) the conclusion of tPA administration: thrombectomy in these cases of AIS with LVO has been considered a rescue therapy after IVT failure’s assessment. These patients presented both a bad clinical outcome (patient 1 died at 15 days for respiratory failure due to pneumonia; patient 2 had a 3-months mRS = 3). Stroke subgroups according to TOAST criteria, baseline NIHSS, ASPECTS scores and occluded vessel were also similar between the two groups (Table 1). Table 2 summarizes the causes of exclusion from IVT. Time-to-groin puncture and time-to-recanalization were similar in the 2 groups. Recanalization rate was significantly higher in CoT group (84.3% vs 65.3%; *P* = 0.009), as well as first-pass success rate (62.7% vs 38.6%, *P* < 0.05). CoT group presented a significantly higher functional independence rate at 3-months follow-up (mRS score 0–1: 48.5% vs 18.6%; *P* < 0.001. mRS score 0–2: 67.1% vs 37.3%; *P* < 0.001).

**Table 1:** Baseline characteristics of the patients

Characteristics	CoT (*n* = 70)	Direct ET (*n* = 75)	*b*
Age, Mean (SD), y	71.9 (10.6)	69.1 (13.2)	0.15^2^
Gender, n F (%)	32 (45.7)	38 (50.6)	0.49^a^
Baseline NIHSS, median (IQR)	18 (15–21)	19 (15–20)	0.55^c^
Risk factors			
Hypertension, n (%)	52 (74.3)	53 (70.6)	0.62^a^
Diabetes mellitus, n (%)	12 (17.1)	11 (14.6)	0.68^a^
Atrial fibrillation, n (%)	30 (42.8)	37 (49.3)	0.43^a^
Hyperlipidemia, n (%)	29 (41.4)	23 (30.6)	0.17^a^
Antiplatelet or anticoagulant use, n (%)	34 (48.6)	37 (49.3)	0.85^a^
Sistolic Blood Pressure, Mean (SD), mmHg	145.6 (12.6)	147.3 (24.8)	0.70^b^
Diastolic Blood Pressure, Mean (SD), mmHg	81.9 (15.3)	81.1 (12.5)	0.77^b^
Serum glucose, Mean (SD), mg/dl	130.4 (54.6)	139.4 (82.7)	0.59^b^
ASPECTS score; median (IQR)	9 (8–10)	9 (8–10)	0.06^c^
Cause of stroke (TOAST)			0.7
Large-artery disease, (%)	23.4	20.0	
Small-artery disease, (%)	0.0	0.0	
Cardioembolic, (%)	54.7	55.0	
Other, (%)	4.7	6.6	
Unknown, (%)	17.2	18.4	
Site of occlusion			
tICA, n (%)	9 (12.8)	22 (29.3)	0.06^a^
MCA-M1 segment, n (%)	46 (65.7)	40 (53.3)	
MCA-M2 segment, n (%)	15 (21.4)	13 (17.3)	

^a^Pearson’s chi-squared test; ^b^Mann-Whitney test for unpaired groups; ^c^Wilcoxon rank sum test; *IQR* inter-quartile range, *NIHSS* National Institute of Health Stroke Scalem, *SD* standard deviation, *MCA* middle cerebral artery, *tICA* terminal ICA, *PCSE* potential cardiac sources of embolism, *CoT* combined therapy, *ET* endovascular therapy

**Table 2:** Causes of IVT exclusion in patients with AIS

Causes of IVT exclusion, (%)	
Anticoagulant therapy	28.6
Brain or systemic lesions at risk of bleeding	14.3
Possible placement of vascular stent	11.4
Onset > 4.5 h	14.3
Major trauma	7.1
Recent surgery	4.3
Other reasons	20.0

*IVT* intravenous thrombolysis

In recanalized subjects, EFIV was significantly smaller in the CoT group (16.4 ml vs 62.3 ml; *P* = 0.003). Safety outcome measures were similar between the two groups (Table 3).

**Table 3:** Details of procedural, clinical, and safety outcomes

Variables	CoT (n = 70)	Direct ET (n = 75)	*P* Value
Time from symptoms onset to needle, Mean (SD), min	156.1 (37.6)	n.a	n.a
Time from symptoms onset to groin puncture, Mean (SD), min	194.1 (59.9)	204.8 (60.4)	0.32^2^
Time from symptoms onset to recanalization, Mean (SD), min	245.9 (75.8)	245.1 (58.6)	0.95^2^
TICI 2b or 3 reperfusion, n (%)	59 (84.3)	49 (65.3)	0.009^1^
Rates of first-pass success, %	62.7	38.6	< 0.05^1^
sICH, n (%)	7 (10.0)	8 (10.6)	0.87^1^
Cerebral infarct Volume, Mean (SD), ml	16.4 (25.3)	62.3 (81.7)	0.003^2^
Outcome at 90 days			
mRS score of 0–1, n (%)	34 (48.5)	14 (18.6)	< 0.001^1^
mRS score of 0–2, n (%)	47 (67.1)	28 (37.3)	< 0.001^1^
Mortality, n (%)	5 (7.1)	11 (14.6)	0.15^1^

^1^Pearson’s chi-squared test; ^2^ Mann-Whitney test for unpaired groups; *CoT* combined therapy, *ET* endovascular therapy, *SD* standard deviation, *ICH* intracerebral hemorrhage, *mRS* modified Rankin Scale, *TICI* thrombolysis in cerebral infarction grading scale

In multivariable analysis, CoT was independently associated with higher odds of favorable functional outcome (OR, 3.75; 95% CI, 1.09–12.85; *P* = 0.03). Other predictors of good outcome were lower baseline NIHSS (OR, 0.73; 95% CI,0.62–0.86; P < 0.05), and vessel recanalization (OR, 7.30; 95% CI, 0.60–88.62; *P* = 0.05) (Table 4).

**Table 4:** Multivariate analysis

Variables	*P* value	OR (95% CI)
Baseline NIHSS	< 0.05	0.73 (0.62–0.86)
ASPECTS score	0.07	0.59 (0.33–1.05)
TICI 2b or 3 reperfusion	0.05	7.30 (0.60–88.62)
CoT treatment	0.03	3.75 (1.09–12.85)
MCA M2 vs. M1 segment	0.15	3.04 (0.66–14.05)
tICA vs. MCA M1 segment	0.25	0.33 (0.05–2.20)
Time from symptoms onset to recanalization	0.11	0.99 (0.98–1.00)
First-pass success	0.15	0.41 (0.12–1.37)

Forward stepwise logistic regression with dependent variable good clinical outcome (MrS score at 90 days: 0–1). *NIHSS* National Institute of Health Stroke Scale, *TICI* thrombolysis in cerebral infarction grading scale, *MCA* middle cerebral artery, *tICA* terminal internal carotid artery, *OR* odd ratio, *mRS* modified Rankin Scale, *ASPECTS* Alberta Stroke Program Early Computed Tomography Score, *CoT* combined therapy

## Discussion

Randomized clinical trials (RCTs) have definitively proven the effectiveness of endovascular approach for anterior circulation AIS with LVO [3]. In this context, the role of adjunctive IVT before ET remains unclear, presenting theoretically pros and cons highlighted in the background section. The main of our study is the independent association between CoT and favorable outcome, compared with direct ET. A higher rate of vessel recanalization and lower infarct volume at follow-up CT seem the plausible biological mechanisms mediating the beneficial effect of CoT on outcome.

Our findings therefore support the use administration of IVT before ET in eligible patients, in line with the recommendations of the American Heart Association / American Stroke Association guidelines [3]. The two excluded patients mentioned in the previous section, received thrombectomy too late after IVT conclusion: this therapeutic scheme different from CoT received by the other included subjects and, because of the short half-life of tPA and is not in line with 2018 guidelines and their inclusion potentially introduce a bias of a delayed ET [3].

The possible reduction of EFIV supports the hypothesis of a direct effect of tPA treatment on potentially salvageable brain tissue. Moreover determination of EFIV may represent a valid outcome measure for future clinical trials, potentially having a higher inter-observer reliability compared to clinical assessment alone with the mRS [12, 13]. The possible suggested mechanisms of action of adjunctive rtPA may be explained by a twofold effect: i) a favorable impact on endovascular procedure as suggested by the higher recanalization and higher first-pass success rates in CoT patients and, ii) an effectiveness of systemic tPA on preserving microvascular perfusion in downstream to the arterial occlusion, therefore improving benefit of large vessel recanalization 4. Finally, another interesting result of our study is the lack of association between tPA and intracranial bleeding. This may have relevant implications for clinical practice, highlighting that tPA treatment in eligible patients should not be withheld for the fear of intracranial hemorrhage. This finding is in line with available evidence [5–11, 18].

Some limitations should be considered in the interpretation of our findings such as relatively small sample size obtained from a single center retrospective analysis, non-randomized comparison, potential risk of confounding by indication due to treatment allocation bias [subjects included in the ET group mostly consisted of patients with contraindications for IVT and therefore possibly weightened by worse prognosis, although the 2 groups are quite homogeneous for clinical and radiological features (see Table 1), lack of advanced neuroimaging-based patients’ selection. We underline that, despite the treatment allocation bias, the two groups are homogeneous regarding the timing of the treatments. This can be explained by the fact that most patients in the ET group did not perform IVT not for exceeding time limit from symptoms onset but for other clinical reasons.

While taking them into full consideration, our data seems to confirm a favorable role of tPA in improving clinical and neuroradiological outcome of patients treated by endovascular mechanical thrombectomy for a large vessel occlusion stroke. The 4 ongoing prospective randomized controlled trials will better clarify this clinical issue.

## Conclusions

The study confirms the safety and beneficial effect of CoT for anterior circulation AIS with LVO compared to direct ET. Coupling EFIV and mRS at 90 days assessment may represent a more reliable and possibly more powerful tool to be used in future clinical trials [11, 12].

## Data Availability

request of data (anonymized dataset) can be done directly to the Corresponding Author (Dr. Massimo Gamba, massimo.gamba@asst-spedalicivili.it).

## Abbreviations

AIS: acute ischemic stroke
ASPECTS: Alberta stroke program early ct score
CIV: Cerebral infarct volume
CoT: Combined therapy
CT: Computed tomography
DSA: Digital subtraction angiography
ECASS: European Cooperative Acute Stroke Study II
EFIV: early follow-up infarct volume
ET: Endovascular therapy
IVT: intravenous thrombolysis
LVO: large vessel occlusion
MCA: middle cerebral artery
mRS: modified Rankin Scale
NIHSS: National Institute Of Health Stroke Scale
RCTs: Randomized clinical trials
sICH: symptomatic intracerebral hemorrhage
tICA: terminal internal carotid artery
TICI: thrombolysis in cerebral infarction
TOAST: Trial of ORG 10172 in acute stroke treatment
tPA: recombinant tissue-plasminogen activator
